# Meaning Construction: Online Narratives of Supervisor-Postgraduate Relationships in Digital Communities

**DOI:** 10.3390/bs16071246

**Published:** 2026-07-21

**Authors:** Jingjing Zhou, Yushu Zhang, Yujie Dong, Lingli Xia, Yuxin Qin, Xiaoli Ye

**Affiliations:** 1 School of Education, South-Central Minzu University, Wuhan 430074, China; zhoujingjing@mail.scuec.edu.cn; 2 Institute of Higher Education, Anhui University, Hefei 230039, China; 3 School of Education, Central China Normal University, Wuhan 430079, China

**Keywords:** supervisor–postgraduate relationship, digital community, meaning construction, sensemaking theory, douban group, netnography, thematic analysis

## Abstract

As digital media become deeply embedded in postgraduate students’ daily learning and social interactions, online narratives of supervisor–postgraduate relationships have emerged as a crucial means for them to express authentic experiences and seek emotional support. Adopting a sensemaking perspective, this study employs online ethnography and thematic analysis to investigate postgraduate students’ narratives of supervisor–postgraduate relationships on a Douban group. The findings reveal four core thematic dimensions of these online narratives: identity formation, emotional interaction, experience sharing, and social connection. These dimensions help postgraduate students clarify their self-perception, release emotions, acquire practical experience, and establish a sense of group belonging, particularly in contexts where institutional support is insufficient. Online narratives essentially serve as a compensatory self-help channel for postgraduate students to cope with real-world challenges. They highlight structural shortcomings in offline supervisory governance and offer theoretical and practical insights to optimize supervisor–postgraduate relationships in the digital age.

## 1. Introduction

The supervisor–postgraduate relationship, as the pivotal link between faculty supervisors and postgraduate students, has attracted extensive scholarly attention amid rapid expansion in higher education and the escalating demand for high-quality talent cultivation (F. Liu et al., 2023; Z. Liu, 2025). Hagstrom (1965) was the first to characterize this relationship as the cornerstone of academic socialization. Building on this foundation, Hudson (2016) further argued that the modern supervisor–postgraduate relationship has moved beyond the traditional master–apprentice hierarchy toward a bidirectional collaborative alliance centered on shared professional goals and sustained by trust, respect, and open, supportive communication. As research progresses, the conceptualization of this relationship has been further expanded and enriched (McKenna & van Schalkwyk, 2024). Grounded in respect, trust, and open communication, it is a multifaceted partnership centered on research and dissertation, encompassing academic guidance, emotional encouragement, collaborative scholarship, and scholarly leadership that foster mutual growth (Ocheja et al., 2025), spanning the entire trajectory of academic development, research training, career planning, and the promotion of physical and mental well-being (Huang & Liu, 2023; Moshtari & Schleper, 2025).

Imbalances and alienation in supervisor–postgraduate relationships are pervasive problems in higher education worldwide, and a series of global surveys published in Nature provide empirical evidence for this phenomenon (Nature, 2025). The 2022 survey, which covered 3253 STEM postgraduate students globally (including participants from China), found that while 65% were satisfied with their supervisor relationship, 22% would choose a different supervisor, 26% would change institution, 45% of bullying perpetrators were supervisors, and only 26% of victims felt safe to speak out (Nature Research, 2022; Woolston, 2022). The 2025 survey, covering 3785 doctoral candidates across 107 countries (including 312 from China), further revealed that nearly half of the students received less than one hour per week of one-on-one communication with their supervisors (Nature Research, 2025). Insufficient supervisory guidance can lead to serious consequences, which is why many universities have established institutional guidelines on supervision frequency and duration. For example, Imperial College London requires supervisors to set aside at least one hour per week for student consultation (Imperial College London, 2025), while Oxford Brookes University adopts “44 h per year (based on one hour per week)” as a guideline for full-time postgraduate students (Oxford Brookes University, 2025). The survey also found that supervisors exhibited significant shortcomings in providing non-academic career guidance and mental-health support; supervisors still accounted for 43% of campus-bullying perpetrators; and only 34% of students recognized their supervisors’ capacity to offer mental-health guidance (Nordling, 2025). These problems not only hinder students’ academic growth but also constrain the overall effectiveness of talent cultivation in higher education (S. Liu et al., 2024).

The challenges that characterize supervisory relationships worldwide are equally pronounced in China’s postgraduate education context. The expanding scale of postgraduate education, bringing increasingly heterogeneous student cohorts and more diverse, individualized demands, has made supervisory interactions markedly more complex (Y. Li, 2021). In China’s expanding postgraduate system, project-oriented management, utilitarian academic culture, and the erosion of traditional supervisor authority have strained and even distorted supervisory relationships, leaving many conflicts unresolved (Zhao et al., 2025). In practice, a number of shortcomings in Chinese supervisor–postgraduate relationships parallel international problems while also reflecting distinctive local features. Some relationships have become tense and distant, displaying obvious authoritarian tendencies and lacking egalitarian communication and emotional support (T. Byrne et al., 2025). Supervisors sometimes fail to provide sufficient academic guidance; in extreme cases, the relationship degenerates into a utilitarian, employer–employee arrangement in which students are tasked with non-academic chores and their legitimate rights are vulnerable to infringement (Hou, 2021). The influence of traditional apprenticeship culture, the utilitarianization of academia, unclear boundaries of power and responsibility, and imbalanced evaluation systems for supervisors have further aggravated the power imbalance (J. Ma & Bie, 2021). Moreover, on-campus mechanisms for appeal, mediation, and psychological support remain underdeveloped, hindering effective prevention and resolution of conflicts (H. Ma et al., 2023). Although Chinese universities and education authorities have continuously refined supervisor evaluation systems, promoted professional ethics, and introduced policies to standardize supervisor–postgraduate interaction, these governance measures largely rely on external constraints and fail to address the deep-seated contradictions of relational alienation and uneven tutoring quality (J. Zhang, 2024), leaving the fundamental dilemmas unresolved.

Rapid technological development in the digital age is continuously reshaping the discursive space available to postgraduate students. China’s 4.2995 million enrolled postgraduate students are predominantly digital natives (Ministry of Education of the People’s Republic of China, 2026), among whom approximately 133,000 are international postgraduate students (Guangming Daily, 2026). This cohort is highly proficient in online communication and tends to convey authentic experiences and emotions concerning supervisor–student relationships within virtual communities (Prensky, 2001). The anonymity and asynchronicity of digital platforms lower expressive inhibitions, creating a safe narrative arena for individuals in vulnerable or sensitive positions (Hinck et al., 2023). From a social-constructionist perspective, identity is co-constructed through interaction and materialized through discursive practice; therefore, online interaction becomes a pivotal medium through which postgraduate students narrate their experiences and develop self-awareness (Delahunty et al., 2014; He, 2024; Naudé, 2023). Consequently, online narration has emerged as a core discursive practice for postgraduate students in the digital era. Because the supervisor–postgraduate relationship spans the entire training trajectory and is routinely beset by concrete conflicts, online discourse predominantly concentrates on overt behavioral misconduct and covert relational alienation (Cheng & Cao, 2019). The anonymity and perceived safety of online narrations provide postgraduates with a space for emotional venting and promote the emergence of virtual communities that blend empathy with instrumental support (Lundeen & Tyler, 2026). Within these communities, the difficulties inherent in supervisor–student relationships can be articulated, examined, and alleviated. Enabled by digital media, such experiences are continually shared, interpreted, and responded to, thereby transforming private conflicts into publicly visible issues that may be resolved (Du & Xu, 2023).

Existing research has redefined the supervisor–postgraduate relationship from multiple perspectives, attempting to depict an idealized picture of interaction (Halse & Bansel, 2012; Heron et al., 2024; Wilmot, 2022). At the same time, it remains grounded in real educational settings, confronting the structural biases and practical contradictions inherent in the operation of the supervisory relationship, and precisely revealing the subtle imbalances, misalignments, and tensions within these interactions (Friedensen et al., 2024; Roos et al., 2021; Xing et al., 2023). Moreover, it thoroughly deconstructs the mechanisms and practical manifestations of disorder in the supervisory relationship, offering diverse interpretive paths for understanding the various forms of alienation emerging in contemporary supervisor–postgraduate relationships (Ives & Rowley, 2005; Sidhu et al., 2014; Yende, 2021). Overall, academic inquiry into the supervisor–postgraduate relationship has formed a relatively mature research trajectory, which has not only deepened systematic understanding of the essential characteristics and operational patterns of these relationships but has also laid a solid theoretical and practical foundation for optimizing postgraduate education models and enhancing the quality of higher education (Xu & Liu, 2023). However, the aforementioned studies have largely focused on offline interaction settings, paying insufficient attention to the transformation of supervisory relationships in the digital age (Tsang, 2025), and have rarely examined the internal mechanisms of online narratives from a subject-centered meaning-making perspective. Some studies have begun to recognize the role of online settings, as instant messaging tools can serve as important extensions of supervisory interaction (Rambe et al., 2024). However, such studies mostly focus on one-on-one private online supervision (Peng & Yu, 2026), paying little attention to postgraduate students’ spontaneous online narratives in anonymous public communities. Unlike private settings, online community narratives are embedded in a collective meaning-making process that includes comments, shares, emotional responses, and other interactive chains (Page, 2018). To address this gap, the present study adopts sensemaking theory as its principal analytical framework and employs online ethnography combined with thematic analysis to conduct a systematic investigation of postgraduate supervisory narrative practices. Specifically, this study addresses the following two research questions:

Q1: What themes of meaning emerge from postgraduate students’ online narratives about the supervisor–postgraduate relationship?
Q2: How do postgraduate students accomplish individual meaning-making through online narratives and interaction?

## 2. Literature Review

### 2.1. Theoretical Development of Supervisor–Postgraduate Relationship Research: From “Apprenticeship” to “Learning Community”

In postgraduate education research and management practice, the supervisor–postgraduate relationship is widely regarded as a core issue (Liang et al., 2021). From a historical perspective, the theoretical interpretation of the supervisor–postgraduate relationship has evolved from an apprenticeship model to a learning community model. Early research tended to view the supervisor–postgraduate relationship as a traditional apprenticeship (Rudolph, 1994), a typical supervisory relationship that emphasizes the supervisor’s role in guiding the student through the research process (Zhou, 2004). Belcher (1994), adopting a traditional apprenticeship perspective, characterized the supervisory relationship as hierarchical and centered on knowledge transmission. In this model, the supervisor serves as a knowledge transmitter and academic guide, while the student gradually acquires research norms and skills under the supervisor’s direction. This model is deeply rooted in the tradition of postgraduate education. Taylor (2012), taking the doctoral education established at the University of Berlin in the early 19th century as a historical benchmark, confirms the master–apprentice model of delivery as the institutional prototype and points out that this model spread and became institutionalized from Germany to the United States, the United Kingdom, and even globally. The core rationale of this model is that the supervisor’s superior knowledge and accumulated experience naturally place him/her in a guiding position, while the student occupies a subordinate apprentice position. Kumar and Stracke (2007) characterize this traditional model as the ‘expert–novice model’, the essential feature of which is that the supervisor, as the expert, guides the novice through the research process.

However, with the evolution of higher education philosophy and the expansion of postgraduate education, this traditional framework has been challenged. Wisker (2012), in her classic work *The Good Supervisor*, points out that the supervisor–postgraduate relationship in modern postgraduate education has moved beyond the apprenticeship model and become more diverse and complex. She emphasizes that an effective supervisory relationship should be a friendly yet professional partnership, maintaining both the professionalism of academic guidance and interaction and emotional support between the supervisor and postgraduate student. At the same time, empirical research has found that the traditional apprenticeship model tends to overlook the broader environmental context of doctoral education and the role that different learning communities play in developing students as independent researchers (Flores-Scott & Nerad, 2012). Some scholars have further proposed the concept of a “supervisor–postgraduate community” (R. Zhang & Ma, 2020). For example, studies have pointed out that the essence of the supervisor–postgraduate relationship is not only a teaching–learning relationship but also a collaborative one (Gillen et al., 2015; Maher et al., 2013). Supervisors and students are equal as individuals, cooperating on the basis of clearly defined rights and obligations, jointly pursuing professional and personal growth (Hong & Hu, 2011). In addition, some scholars have introduced the concept of a learning community, situating the supervisory relationship within a broader social network. For instance, J. Li et al. (2020) define the learning community as “a new type of partnership formed between supervisors and postgraduate students through social interaction based on shared research visions, life values, and emotional bonds”. From this perspective, students’ academic growth depends not only on their individual interactions with the supervisor but also on peer groups, academic networks, and the broader academic community. As Nerad (2012) points out, a paradigm shift is currently taking place in doctoral programs worldwide, moving from one-on-one, top-down master–apprentice transmission to structured learning conducted within multi-level learning communities both inside and outside the university, a process in which peers play a crucial role. A. L. Byrne et al. (2026) similarly point out that supervision in higher degree research has “historically followed a master–apprentice model, privileging a dyadic relationship”, but that cohort supervision now “represents a move away from traditional dyadic supervision models to a more inclusive collaborative design”. This theoretical shift redefines the roles within the supervisor–postgraduate relationship, providing an important explanatory framework for understanding student interactions in online communities and their significance for the supervisory relationship. It also offers a starting point for clarifying the core debates in existing research.

### 2.2. Core Issues in the Supervisor–Postgraduate Relationship: Power, Emotion, and Identity

In international research on supervisor–postgraduate relationships, power, emotion, and identity are three intertwined core issues that provide important analytical dimensions for understanding the complex nature of the supervisory relationship (Bao et al., 2025; Berge & Kobayashi, 2026; Y. Zhang et al., 2025). Power structure is one of the earliest and most debated issues in this field. For example, Manathunga (2007) points out that even when the supervisory relationship is presented as an egalitarian apprenticeship, it is always embedded in inescapable power dynamics and governance logic. From a critical perspective, Grant (2019) revealed the multiple dimensions of power in the supervisory relationship, and in her subsequent work (Grant, 2024) identified four interacting domains of power, namely governance, pedagogical, interpersonal relationships, and desires, which shows that power in the supervisory relationship is not a unidimensional relationship of domination and subordination but a complex phenomenon. Thus, power can manifest as explicit control and discipline or can implicitly permeate the subtleties of daily interactions between supervisor and student. Research focusing on the causes of problems in the supervisory relationship points to a deeply entrenched power imbalance between postgraduate students and their supervisors (Z. Liu, 2020; H. Ma et al., 2023). Notably, Tsang (2025), drawing on Bourdieu’s concept of symbolic power, argues that the shimen in the Chinese academic field further reinforces this asymmetrical power structure, in which supervisors, relying on their academic resources, their control over graduation decisions, and their cultural authority, exercise a form of legitimate domination, compelling students to remain silent under oppression, even when facing boundary violations. Similarly, Xing et al. (2024) found that research students were exposed to three forms of symbolic violence: supervisor authority, English hegemony, and neoliberalism.

In recent years, as attention to postgraduate students’ mental health has increased, the emotional dimension of the supervisory relationship has become a key research focus (Mavrogalou-Foti et al., 2024). Studies show that supervisors, as key figures affecting students’ academic trajectories, play a crucial role in creating an interactive emotional atmosphere and defining boundaries (Wollast et al., 2023). The supervisor–postgraduate relationship is not merely a process of academic knowledge transmission and cognitive guidance, but a social practice involving emotional interaction, emotional investment, and emotional labor (Hu et al., 2023). For example, Nachatar Singh (2022), a study of international postgraduate students showed that high-quality supervisory relationships are long-term and sustainable, extending beyond graduation as manifested in ongoing research collaboration and career development support. From the supervisor’s perspective, through qualitative interviews with 19 supervisors, Knox et al. (2006) found that in good supervisory relationships, supervisors experience strong personal satisfaction, such as joy of working with students who are interested in learning, discovering, and exploring new areas, while in difficult relationships, they incur significant emotional costs, including frustration, anxiety, and exhaustion, with some even adopt negative coping strategies, such as praying for their advisee’s graduation as an end to their conflict. Pyhältö et al. (2015), from the perspective of both students and supervisors, found that emotional support, constructive feedback, and collaborative thinking are central to the supervisor’s mentoring tasks, and that supervisors who show respect, offer encouragement, and provide an open communication environment are key to a high-quality supervisory relationship, significantly enhancing students’ satisfaction with supervision. J. Han and Jin (2025) reveal that the complex emotions experienced by postgraduate students in daily interactions with their supervisors accumulate through an interaction ritual chain, forming positive or negative emotional energy. This energy not only directly affects students’ academic efficiency and outcomes but also shapes the strength of social bonds between supervisor and student, influencing students’ academic identity and development trajectories. Thus, emotional interaction has become the core adhesive that either sustains or dissolves the supervisory relationship, serving as both a positive driver for mutual growth and a key trigger for conflict and breakdown (Y. Han & Xu, 2021; Jin et al., 2026).

The issue of identity is closely related to power and emotion in the supervisory relationship. Postgraduate study is not only a process of acquiring knowledge but also a process of self-cultivation and personal transformation (Green & Lee, 1995). Thus, the supervisory relationship is essentially a process of constructing the postgraduate student’s academic identity. Petersen (2007), drawing on the concept of subjectification, argues that in the supervisory relationship, postgraduate students gradually internalize what counts as culturally intelligible academic performativity through repeated “category boundary work,” thereby becoming appropriate academic subjects. Further research points out that the supervisory relationship is an important arena for postgraduate students’ academic identity formation, where the alignment between the supervisor’s cognitive schema of the ideal researcher and the student’s self-perception significantly affects the student’s experience of supervision and their sense of academic belonging (Janssen et al., 2021). By participating in research projects under the supervisor’s guidance, receiving feedback on academic writing, and gaining recognition within the academic community, postgraduate students move toward the completion of their academic identity construction and academic socialization (Bao et al., 2025). However, research also reveals that in extreme cases, the supervisory relationship can become a “warzone of identity,” where students experience alienated selves such as the “surveilled self,” the “overwritten self,” and the “censored self” (T. Byrne et al., 2025). This identity transformation process is not linear but dynamic, full of negotiation, adaptation, and even conflict. Postgraduate students need not only to find a balance between their supervisors’ expectations and their own self-positioning, but also to achieve identity integration across multiple roles, such as learner, researcher, and laborer (Sun & Li, 2023).

### 2.3. Influencing Factors and Problem Domains in the Supervisor–Postgraduate Relationship

In fact, the supervisor–postgraduate relationship is not only a personal connection but also closely linked to the cultivation of students’ academic research abilities, the achievement of their personal academic goals, and the realization of the teaching and research goals of the university and supervisor (S. Wu et al., 2024). Moreover, the formation and development of the supervisory relationship are influenced by multiple factors. Existing research mainly analyzes these factors from three levels: individual characteristics, interpersonal interaction, and social environment (Sidhu et al., 2014; W. Wu et al., 2024a; Yende, 2021).

At the level of individual characteristics, supervisors and postgraduate students are two groups with heterogeneous traits within the same research field, and the supervisory relationship is inevitably influenced by the overall qualities and individual characteristics of both parties (W. Wu et al., 2024b). Personal traits of both supervisors and students have been shown to significantly affect the supervisory relationship. For example, Mainhard et al. (2009) operationalized supervisors’ interpersonal communication styles into two core dimensions, namely influence and proximity, and derived eight specific types, including leadership, helpful, friendly, understanding, and others. Research has shown that different supervisory styles elicit different interactive responses and relational experiences from students (Song & Mei, 2012). Sinclair (2004), based on a national survey in Australia, found that two different supervisory styles, interventionist and laissez-faire, significantly affect doctoral students’ progress, degree completion, and interaction experiences. Furthermore, Denicolo (2004) reported that from the student’s perspective, supervisors should possess positive traits such as reliability, confidence in the student, encouragement, being knowledgeable, being informative, and willingness to share. Likewise, students’ initiative, communication skills, and emotion regulation strategies play important roles in the formation of the supervisory relationship. Ives and Rowley (2005) found that a good interpersonal working relationship between supervisor and student is closely related to student progress and satisfaction, and that the establishment of the supervisory relationship depends not only on the supervisor’s style but also to a large extent on the student’s willingness to actively communicate and engage in interaction.

At the level of interpersonal interaction, factors such as the frequency of communication, depth of interaction, and expectation alignment have become the focus of research. During the process of receiving supervision, the supervisor’s task allocation, communication attitude, daily interaction, and understanding of and support for the student all affect the development of the supervisory relationship (Chen & Hu, 2022; Le et al., 2021). Inman et al. (2011) demonstrated the importance of expectation alignment from the perspective of nondisclosure behavior. Their study found that expectations by advisors that were unclear/mixed/unreasonable were the most frequently concealed issue from supervisors by doctoral students, and such concealment was significantly negatively correlated with lower satisfaction with supervision. Thus, when students perceive an expectation mismatch with their supervisor, they are more likely to avoid disclosing their confusion or problems for fear of damaging the relationship or facing negative evaluation, thereby weakening the quality of the supervisory relationship.

At the level of social environment, factors such as disciplinary culture, departmental climate, and institutional arrangements constitute the macro context in which the supervisory relationship unfolds. Different disciplines, due to differences in their knowledge production methods and academic cultures, have formed distinctive patterns of supervisory relationships (Chiang, 2003). Science and engineering disciplines often emphasize teamwork and laboratory culture, resulting in a stronger collective and instrumental character in the supervisory relationship, while the humanities and social sciences focus more on independent research and intellectual dialogue, making the supervisory relationship more individual and affective. For example, Emke-Francis (2010), studying clinical psychology doctoral students, found that the research laboratory and the department were contextual forces affecting the supervisory relationship. The research laboratory influenced the relationship by giving supervisors the dual roles of team leader and employer, and by introducing peer competition, comparison, and support networks; the department influenced supervisor–postgraduate interaction by providing institutional support and acting as an evaluative body. These findings indicate that the supervisory relationship does not exist in isolation but is embedded in a multi-layered network of social contexts.

## 3. Theory Basis

The sensemaking theory, originally articulated by Dervin (1983), posits that individuals, when confronted with uncertainty, actively bridge cognitive gaps by constructing subjective meanings through information-seeking, narrative expression, and social interaction. This process enables them to comprehend their circumstances, regulate emotions, and guide subsequent actions. Dervin’s “Situation–Gap–Use” analytical framework treats meaning not as an objective given but as a dynamic product generated through dialogue, reflection, and sharing within particular temporal, spatial, and social contexts (Turner et al., 2023). The theory rests on three core tenets. First, reality is characterized by discontinuity and uncertainty. As Dervin (1998) posited, sensemaking originates at the confrontation of a “‘gappyness’ or a discontinuity” in the world. When individuals experience cognitive confusion or action dilemmas, they develop “cognitive gaps” that motivate information-seeking and meaning-making behaviors. Second, humans are active meaning-makers. Dervin (1992) emphasized that information is not something that exists independently, but is “created at a specific moment in time-space by one or more humans”. Third, meaning is contextual and social. Dervin (1999) argued that individuals construct “interpretive bridges over a gappy reality”, a process that is co-constructed within specific contexts. Through group interaction, individuals develop emotional resonance and collective identities, which generate shared meanings and support systems. As a classic theory that foregrounds the information behaviors and meaning production of actors, sensemaking theory offers a stable analytical lens for examining how individuals achieve cognitive repair, emotional regulation, and social connection through interaction and narrative in the face of dilemmas. Consequently, it provides a robust theoretical foundation for the present study’s investigation of the value-generation mechanisms underlying online narratives.

This study adopts sensemaking theory as its analytical lens and follows the logical progression of uncertain situations, cognitive gaps, online narrative practices, and meaning generation. The relational tensions, communication barriers, and insufficient support that postgraduate students encounter in supervisor–postgraduate interactions constitute a prototypical uncertain situation that triggers meaning construction. These circumstances give rise to pronounced gaps at the cognitive, affective, and behavioral levels, prompting postgraduate students to seek information and outlets for self-expression in online spaces. Digital communities then provide a platform for narration, interaction, and experience sharing, enabling students to bridge these gaps through information-seeking and narrative activities. Ultimately, through sustained online narration and collective interaction, postgraduates achieve meaning construction both individually and collectively. Grounded in the core logic of sensemaking theory, this framework highlights the mechanisms and processual features of online narration within supervisory relationships, offering a clear and systematic approach to interpreting the internal logic of postgraduate students’ online narratives.

## 4. Methods

### 4.1. Research Design

This study adopts a qualitative research design combining netnography and thematic analysis. Netnography, a qualitative research method (Kozinets, 1997), integrates participant observation and lurking observation to collect online data within the Internet context. Compared to traditional interviews or focus group discussions, it offers greater naturalness, nuance, and immersion. Today, conceptualizing online social platforms as digital fields has become a scholarly consensus (Costello et al., 2017; Hardcastle et al., 2019). In traditional social science research, respondents tend to provide socially desirable answers to gain higher social approval (Sheng & Shi, 2024). The most prominent advantage of netnography, however, is that it allows researchers to capture naturally occurring conversations (Björk & Kauppinen-Räisänen, 2012), avoiding the social desirability bias that may arise in offline interviews. It enables access to respondents’ authentic, unfiltered attitudes and experiences in real settings, making it particularly suitable for exploring sensitive issues such as supervisor–postgraduate conflict, topics that are difficult to discuss openly or face-to-face. Therefore, this study focuses on the Douban discussion group “Has your supervisor cared about you today?” Douban (douban.com) is a well-known Chinese cultural community platform that gathers a large number of young users, primarily university students, postgraduates, and young intellectuals. The discussion group was established in 2019. As of August 2024, it had attracted over 200,000 postgraduate members from various disciplines both in China and abroad, and its membership continues to grow. Using netnography, we capture postgraduates’ emotional expressions, experiential writing, and interaction patterns in their everyday state, thereby observing how they narrate the joys and sorrows of the supervisor–postgraduate relationship in a relatively free and anonymous online space, how they form emotional resonance and experiential connections through replies and comments, and ultimately present the overall picture and cultural logic of online narratives about the supervisory relationship.

However, given the highly individualized and diverse narrative structures that postgraduates display when expressing opinions and personal experiences, traditional statistical and analytical methods cannot fully capture the complexity. Therefore, this study supplements netnography with a more fine-grained textual thematic analysis as a tool for data processing and interpretation. Thematic analysis is a qualitative method for identifying, analyzing, organizing, and reporting recurring patterns of meaning in data (Aronson, 1994). It emphasizes systematic coding to progressively distill themes from raw texts, maintaining both fidelity to the data and openness, while establishing a clear and traceable analytical chain. The combination of netnography and thematic analysis preserves sensitivity to postgraduates’ authentic expressions while ensuring systematic analysis and theoretical rigor.

### 4.2. Research Field and Participants

#### 4.2.1. Research Field

The research field of this study is the Douban discussion group, “Has your supervisor cared about you today?”. The discussion group was established in 2019. As of August 2024, it had attracted over 200,000 postgraduate members from various disciplines both in China and abroad, and its membership continues to grow. The group’s description explicitly states: “University teacher–student relationships have recently been pushed to the forefront. Come here to chat about your daily interactions with your supervisor. Let’s admire caring supervisors together and rant about bizarre professors.” This statement clearly reflects the core concerns of group members: sharing real experiences and emotions about the supervisory relationship in a relatively relaxed and anonymous space.

To facilitate discussion, the group has several thematic sections, including “Loud Confession” for sharing supervisors’ warm-hearted behavior and effective guidance; “Open Rant” for expressing dissatisfaction, confusion, or conflict in the supervisory relationship; “Question Time” for seeking advice on specific problems such as communication strategies or thesis guidance conflicts; and “Veterans’ Speak,” where alumni or experienced senior postgraduates share reflections and lessons. These sections give the narratives within the group a strong thematic focus and structural coherence. Posts typically contain clear narrative threads, vividly portraying the real picture of interactions between supervisors and postgraduates.

In addition, the discussion group enforces strict review mechanisms for membership applications, posts, and comment content. New members must apply to join, posting and commenting must follow group rules, and discussions are limited to topics related to the supervisory relationship. This clearly bounded, semi-private spatial design enhances the observability and focus of topics, ensures a reasonable degree of data reliability, and provides an ideal online field for this study.

#### 4.2.2. Participants and Sample

This study follows the principle of purposive sampling in qualitative research, focusing on postgraduates who actively write narratives about the supervisory relationship in the group. Sample selection was based on three criteria. First, topic relevance: all posts included in the analysis must revolve around daily supervisor–postgraduate interactions, with complete narrative content that clearly presents the author’s situation, emotions, cognitions, or action intentions. Second, narrative completeness: following the logic of “key case sampling” in qualitative research, only posts with relatively complete narrative structures were selected. Third, for heterogeneity coverage, to ensure that the sample reflects narrative differences among postgraduates from diverse backgrounds, attention was paid to covering a variety of educational levels, disciplines, and narrative types. This helps avoid over-concentration on a particular group or a single narrative tendency. Based on the above sampling criteria, after data crawling and manual screening, the researchers ultimately identified 260 posts that provide complete narratives of the supervisory relationship and 1326 valid comments as the core analytical sample.

### 4.3. Ethics

This study adheres to the basic ethical principles of qualitative research, with particular attention to ethical issues in digital fields as online social platforms and communities become increasingly prevalent (Busher & Fox, 2019). In the Douban discussion group “Has your supervisor cared about you today?”, a semi-public online community of over 200,000 members, we faced ethical challenges regarding the ambiguous public–private boundary of the online space and the practical difficulties of obtaining informed consent from all members. Given the large size of the community and the fragmented nature of interactions, obtaining prior written consent from every member was both impractical and likely to undermine the naturalness of the research (Kozinets, 2015). Langer and Beckman (2005) point out that the key criterion for determining whether online content belongs to the public or private domain is access restriction: if access is restricted by a password (in addition to the platform login), the content can be considered private; conversely, if only platform registration is required for access, it can be considered public. Although membership in the Douban group under study requires an application, once admitted, all posts and comments are visible to all group members and can be browsed and retrieved without additional passwords. Thus, it can be defined as a semi-public space. Moreover, users’ core motivation for participating in the group is to openly share personal experiences and emotions about the supervisory relationship, as the group description explicitly encourages members to “chat about your daily interactions with your supervisor.” This expressed intention further reinforces the public nature of the content. Following the ethical approach adopted by netnographers in public spaces and drawing on traditional ethnographic principles for observing public behavior, this study employed a direct, low-intervention netnographic method. To protect participants’ privacy, all quoted posts and comments were strictly de-identified: usernames, profile pictures, links, and other identifying information were removed, and details that might reveal participants’ universities, disciplines, or supervisors were paraphrased or obscured to ensure that readers cannot trace back to participants’ real identities. This study did not collect any private communications or posts marked as “do not quote.” The study was approved by the Institutional Review Board of Anhui University (Approval No.: AHUIHE20231207).

### 4.4. Data Collection

Before formally starting the observational study, to effectively mitigate the limitations of the “information cocoon” effect and potential cognitive biases caused by unclear discourse patterns, the researchers first conducted a preliminary lurking observation. During this phase, the researchers, as non-participant “observers,” familiarized themselves with and internalized the cultural context, communication rules, and interaction norms of the discussion group. They broadly browsed content across various sections of the group without preset goals, aiming to construct an initial cognitive framework of the narrative content, expression styles, and interaction patterns related to the supervisory relationship within the group.

In the preliminary phase, the researchers entered the group as ordinary registered users to familiarize themselves with the community’s cultural context and interaction norms without intervening in the group’s natural discourse. Subsequently, starting in January 2024, the researchers formally joined the group as participating members with their researcher identities disclosed in their profile bio, adopted an immersive stance, and began a nine-month period of deep participant observation. To ensure the authenticity and immersion of participant observation, the researchers maintained an appropriate psychological boundary while actively engaging in the discussion atmosphere, initiating and participating in conversations, and building positive, interactive relationships with members. By carefully reading thematic posts such as “My daily life with my supervisor,” “What should I do?” and “A guide to supervisor–postgraduate interaction,” and meticulously documenting the comment interactions and feedback mechanisms among members, the researchers sought to comprehensively capture and understand the diverse themes, emotional depths, and cognitive differences displayed in online narratives about the supervisory relationship by members with different educational backgrounds and personal experiences.

After the observation period, to further explore the value behind the data, the researchers used data crawling techniques to accurately retrieve all posts in the discussion group from 6 January 2023 to 31 August 2024. To further ensure the accuracy and reliability of the study, the screened posts were systematically numbered, each assigned a unique identifier from “DB001” to “DB260.” This numbering system facilitates rapid location and citation of specific posts during the research process and supports subsequent data coding.

### 4.5. Data Analysis

This study used NVivo 12 qualitative data analysis software to conduct thematic analysis on the posts and comments collected from the Douban discussion group. The analysis employed both deductive and inductive coding approaches to effectively answer the research question ”How do postgraduates construct meaning about the supervisory relationship through narratives in online communities?” and to systematically present the four core dimensions of meaning-making. Specifically, deductive (top-down) coding was based on the analytical framework of sensemaking theory, focusing on the uncertain situations described by posters, the perceived cognitive and emotional gaps, and the uses of meaning achieved through online narratives (e.g., identity formation, emotional release, experience acquisition, and community connection). Inductive (bottom-up) coding was derived from the raw textual content of the 260 posts and 1326 comments through progressive abstraction, thereby achieving integration and mutual validation of the theoretical framework and empirical data (see Table 1). Two coders independently performed content analysis on the 260 selected posts, categorizing them into corresponding coding categories according to the narrative themes about the supervisory relationship mentioned in the posts, to ensure coding accuracy. After independent coding, the researchers compared the two coders’ results. Discrepancies were resolved through discussion and analysis of their causes, ultimately reaching consensus.

### 4.6. Rigor and Trustworthiness

To ensure the rigor of this netnographic inquiry, we strictly adhered to the four quality criteria proposed by Kurikko and Tuominen (2012). In terms of immersive depth, we conducted a nine-month participatory observation, collecting multiple sources of data, including narrative posts, comment threads, and reflexive field notes, and cross-validating these to gain an in-depth understanding of the community culture. Prolonged engagement was manifested through the sustained fieldwork period, which allowed us to track the evolution of topics and the dynamics of interaction patterns, ensuring that the research was grounded in authentic practice. Regarding researcher identification, we conducted a preliminary period of lurking observation to familiarize ourselves with the community context before formally entering the field, after which we disclosed our researcher identities in our profile bios and began formal fieldwork. Throughout the process, we continuously reflected on our dual roles as both “observers” and “active participants,” while strictly implementing anonymization and ethical disclosure. Persistent conversation was reflected not only in our active participation in community discussions but also extended to the data analysis phase where two coders independently performed thematic analysis on the selected texts, and all coding discrepancies were resolved through repeated discussion and negotiation until consensus was reached. This process itself embodied the ongoing dialogue between researchers and between researchers and the data, thereby ensuring the robustness of the analytical findings.

## 5. Findings

In the context of the rapid proliferation of social media platforms, postgraduate students employed online narratives to convey a candid account of their interactions with supervisors. From these narratives, four interrelated dimensions of the supervisor–postgraduate relationship emerged, namely identity formation, emotional interaction, experience sharing, and social connection. Together, they illustrated how online narration, as an essential meaning-making practice, supported postgraduates in developing self-awareness, regulating affect, and rebuilding relational ties when faced with the challenges inherent in the supervisor–postgraduate dynamic.

### 5.1. Dialogue with the Self: Identity Formation in Experiential Writing

Narrative is a central means through which humans understand themselves and the world, and it also serves as a key mechanism by which identity is shaped and performed in personal, social, and digital contexts (Hnit & Almanna, 2025). This aligned closely with sensemaking theory: identity is not predetermined but is gradually constructed through the interpretation of experiences (Weick et al., 2005), and online narrative provided an open and safe space for this process. In their interactions with supervisors, postgraduate students often faced the dilemma of multiple roles, blurred boundaries, and uncertain paths of identity and growth; this uncertainty drove them to engage in sensemaking through online narratives. At its core, online narratives about the supervisor–postgraduate relationship were a form of self-dialogue that postgraduates conduct in the digital space. It not only records their experiences within this relationship but also fosters a stable sense of self through the process of reflecting on and organizing those experiences. Through this process, postgraduates reflected on their multiple roles within the supervisory relationship, achieved academic socialization through continuous expression and interaction, and deepened their sense of identity. In practice, this reflective process took shape in two distinct ways: identity construction and self-reflection.

#### 5.1.1. Identity Construction: “I Am a Postgraduate Student”

In daily supervisory interactions, postgraduate students faced persistent practical challenges, including role ambiguity, unclear academic positioning, and the difficulty of aligning their self-expectations with their supervisors’ requirements. Some participants captured this uncertainty bluntly. One wrote: “No matter how well I prepared my reports, in my supervisor’s eyes I was good for nothing, and she could never be satisfied” (DB204). Others voiced similar frustrations of receiving relentless criticism on their academic work over the years: “At first my supervisor was polite and encouraging, but as I proved to have weak foundational knowledge and little research experience, his attitude toward me changed completely” (DB168). Students experienced significant uncertainty in their identity perception and role confirmation, creating an urgent need to engage in narrative practices that helped them construct meaning and clarify, solidify, and anchor their internal identity. One participant traced her motivation for posting directly to this distress: “I had no one around me who could truly understand my academic distress, so I chose to share my story online” (DB227). Another framed the act of writing as a form of emotional relief and self-healing: “Recording my painful academic journey helps me rationalize my confused identity and uneasy emotions” (DB201). A postgraduate student’s academic identity was not pre-existing; rather, it was continuously interpreted and gradually constructed through research practice, mentoring interactions, and feedback dialogues. Their role perception and self-understanding were constantly deepened through concrete, tangible supervisory behaviors. Narratives related to the supervisor–postgraduate relationship typically centered on specific supervisory guidance, such as research project collaboration, academic paper writing and revision, and feedback communication (W. Wu et al., 2024b). These real-life scenarios served as crucial material for sensemaking and identity formation. Through systematic reflection on their interactions with supervisors, postgraduates continually examined their position, responsibilities, commitments, and actual contributions in academic research. By reviewing their experiences and interpreting their meanings, they gradually clarified their self-positioning and role perception, thereby establishing a stable, clear, and internally affirmed postgraduate student identity. This identity construction was not achieved overnight but unfolded through a continuous process of evolution, moving through pre-construction, real-world adaptation, and identity confirmation. In the early stages of enrollment, based on ideals and aspirations, students formed a forward-looking conception of their future academic role, which constituted identity pre-construction. Upon entering actual academic training, the ideal identity encountered real-world pressures, dilemmas, and challenges. The gap between expectation and reality was starkly expressed by one participant: “I once anticipated a rigorous and rewarding academic life, but continuous supervisory setbacks reshaped my initial imagination of postgraduate research” (DB145). Self-awareness awakened amidst these conflicts and underwent dynamic adjustments, a process that marked reality adaptation. Finally, through continuous online narratives and peer interactions, the identity became grounded and solidified, thereby achieving identity confirmation. Postgraduates continually affirmed their self-identity through narrative, as reflected in their aspirations for their future academic path: “to become a researcher with outstanding research capabilities and substantial academic contributions, just like my supervisor” (DB150); in their perseverance in academic beliefs despite adversity: “No matter how rugged the path of postgraduate study may be, I must persevere until the dawn of victory arrives” (DB108); and in their reflective definition of their growth trajectory: “Countless nights with the lights on, countless experimental results rejected by my supervisor, and countless resolutions to start over—these pieces come together to form my postgraduate school life” (DB152).

#### 5.1.2. Self-Reflection: “Personal Growth Records”

In the dynamic process of establishing and mutually adapting to the supervisor–postgraduate relationship, postgraduates often encounter practical challenges, including adjustment difficulties, communication barriers, cognitive confusion, and uncertainty about their developmental direction (Chugh et al., 2022; Y. Li et al., 2025). Traditional settings often lacked safe, open spaces for expression, whereas online narrative writing provided a low-pressure, highly inclusive, and sustainable medium for self-reflection that supports meaning construction amid uncertainty. Based on sensemaking theory, self-reflection in the supervisory context is not merely a retrospective review of experiences but rather a deep metacognitive activity aimed at self-improvement, which is a crucial step in postgraduate students’ active construction of meaning. Narrative writing enabled postgraduates to step outside the “insider’s” perspective and, in the dual roles of “recorder” and “reflector,” critically examined their academic journey, supervisory interactions, emotional experiences, and personal growth. The dual role was vividly captured by one participant: “I write down all my struggles and confusions to sort out my experience and reconcile myself with my tough postgraduate life” (DB179). Through such writing, participants moved from passive suffering to active reflection. It allowed them to systematically analyze the challenges and opportunities, efforts and rewards, as well as the weaknesses and strengths encountered during their development. On the one hand, online documentation allowed postgraduates to confront their shortcomings and practical difficulties in supervisory interactions, accurately identifying issues such as communication barriers, differences in interdisciplinary backgrounds, unclear goals, and a lack of initiative. It also enabled them to express their inner needs, confusions, and expectations clearly and authentically, with one student admitting: “My interdisciplinary background makes it very hard for me to grasp what my supervisor is trying to say” (DB217), and another confessing: “I don’t want to spend three years in a daze” (DB197). On the other hand, systematic and sustained reflection helped postgraduate students clarify their personal traits, strengthen their self-concept, and enhance their sense of self-efficacy. While acknowledging their shortcomings, they also built confidence and internal motivation to navigate the supervisory relationship, as evidenced by comments such as, “I am indeed too introverted; I really need to be more proactive in communicating with my supervisor” (DB101). Amid the intertwined experiences of pressure and growth, postgraduate students also reinterpreted the value of the supervisory relationship, constructing deeper meanings: “Under my supervisor’s strict demands, I often feel immense pressure, but I also know very clearly that if I hadn’t met such a great teacher, I wouldn’t have grown along the way to become the better person I am today. I hope to live up to my supervisor’s expectations and continue to improve” (DB135).

### 5.2. Co-Presence Feeling: Emotional Aggregation During Online Carnival

In real-world supervisory interactions, postgraduate students commonly faced challenges such as a lack of emotional support, an inability to vent negative emotions, and persistent loneliness (Ore, 2021). This tended to create a pronounced emotional–cognitive gap: students found it difficult to express their true feelings openly in formal settings and struggled to receive timely responses or deep empathy from their supervisors or peers, resulting in prolonged emotional suppression and weak social connections. Digital communities, with their open, diverse, and low-risk discourse environment, transcended the constraints of face-to-face expression and power structures, providing a vital space for the dissemination of supervisor–postgraduate topics and emotional resonance. As a young cohort whose values are still being shaped, postgraduates had a strong need for emotional release, affective exchange, and a sense of belonging. Social media could effectively promote peer interaction and strengthen group participation and belonging (Santoveña Casal, 2019). Given this context, online narratives of the supervisor–postgraduate relationship not only served as a stable outlet for postgraduate students’ emotional release but also fostered spiritual resonance through peer interaction, thereby helping individuals construct meaning and achieve psychological adjustment at the emotional level.

#### 5.2.1. Emotional Expression: “Internet Tree Hole”

In real-world academic settings, postgraduate students often find themselves trapped in unresolved emotions and mounting academic pressures with no clear outlet. Digital communities, bound by shared interests, consequently became important spaces for emotional expression, psychological adjustment, and meaning construction. For one thing, leveraging their community-based connections and open participation, online platforms could effectively alleviate postgraduates’ feelings of academic isolation and stress, allowing them to openly share their academic struggles, research anxieties, and educational experiences with peers (Le Busque & Mingoia, 2023). For another thing, vertical academic communities featured focused topics and homogeneous groups, where members easily formed consensus around issues related to the supervisor–postgraduate relationship and received immediate collective responses, thereby providing a communal context for individual emotional interpretation and meaning-making. In virtual interactions, emotions served as a core driving force behind information flow, topic dissemination, and group cohesion, profoundly influencing the reach of narratives and group reactions, and acting as an intrinsic driver of postgraduates’ collective emotional meaning-making. The communicative efficacy of online narratives about the supervisor–postgraduate relationship was closely tied to their emotional tone; different emotional expressions gave rise to differentiated group identities and meaning generation. Positive narratives could unite and convey beneficial emotions such as optimism and approval, fostering a supportive community atmosphere. One commenter wrote: “It is such a great blessing to meet a supervisor who is truly considerate and supportive of students” (DB196). Members conveyed recognition and encouragement through comments such as “envy” and “congratulations,” transforming the community into a vehicle for aggregating collective emotions and transmitting positive values, thereby achieving positive meaning construction. Another member commented: “Congratulations to the original poster! It’s a blessing to encounter a good supervisor. By sharing this experience, you’ve made me look forward to meeting such a person myself and inspired me to become one too” (DB250). Compared to positive narratives, negative emotions typically carry greater infectious power and resonance in online spaces. One participant wrote: “I have tried my best on every task, yet I never receive a single word of affirmation from my supervisor” (DB177). Another expressed: “Three years of postgraduate life has been nothing but suffering and self-doubt” (DB169). High-intensity emotional expressions such as “agony,” “breakdown,” and “internal conflict” easily triggered emotional resonance and mutual support among peers. In sections like “Open Mic Rants,” a collective atmosphere of empathy, understanding, and comfort generally took shape within the community. Members engaged in emotional interaction through likes and comments, jointly interpreting supervisory challenges and building a shared sense of values through the exchange of experiences and shared empathy. One participant noted: “Speaking it out made me feel a bit better” (DB129). Through ritualized group emotional expression and discursive practices, they achieve emotional resolution and meaning reconstruction for individual negative experiences, forming a typical pattern of online collective interaction. Another participant reflected: “After releasing negative emotions, I can calmly view my academic setbacks and adjust my research strategy rationally” (DB134).

#### 5.2.2. Emotional Resonance: “Community of Shared Destiny”

The multiple challenges encountered in real-world supervisory interactions could easily leave postgraduate students feeling helpless, isolated, and plagued by self-doubt, further exacerbating emotional distress characterized by alienation, lack of support, and a sense of being misunderstood (White et al., 2024). Many students experience persistent negative feedback from supervisors, with one participant describing pervasive self-doubt: “I was left feeling completely unrecognized and worthless in my supervisor’s eyes” (DB165). This drove them to turn to relatively safe online spaces in search of a sounding board, comfort, and a sense of belonging. Compared to strong-tie social platforms such as Weibo and WeChat Moments, Douban groups possessed unique attributes of weak socialization, high privacy, and controlled access. Bonded by shared interests and characterized by weak ties, they could significantly reduce postgraduates’ social pressure, relational concerns, and the burden of self-expression (Dong & Zhu, 2024). The private, low-pressure environment enables authentic self-disclosure without social restraint, as one user noted: “I can speak my true thoughts without worrying about judgment from acquaintances” (DB139). Through strict application mechanisms and group rules, these groups established relatively closed, clearly bounded spaces for communication. This effectively shielded members from the “gaze of others” and evaluative interference from external perspectives, providing a safe space for individuals to disclose their true selves, share experiences, and interpret situations. Postgraduate students facing various academic and supervisory challenges were able to candidly share their real-life experiences and inner struggles. As one poster candidly stated: “If it weren’t for this discussion group, these words would just rot inside me” (DB121). Decentralized digital community interactions dissolved hierarchical identity differences; members assumed dual roles as both speakers and listeners. Through equal dialogue and continuous responses, they built a shared understanding of their circumstances, established emotional connections, and formed a “community of shared destiny” in the face of supervisory challenges. The real-time interactions in the comment section fostered a sense of online co-presence that transcended time and space, mitigating real-world social barriers. Individual narratives could quickly receive empathetic responses and experiential references. This efficient, empathetic, and inclusive feedback mechanism significantly stimulated postgraduates’ willingness to express their emotions, encouraging them to proactively share their supervisory dilemmas, stress experiences, and personal insights in the online space. The cross-temporal emotional connections formed among individuals based on shared difficulties effectively bridged the cognitive gap stemming from loneliness, helplessness, and alienation, providing postgraduates with vital psychological support and a sense of meaning. One postgraduate student remarked: “I feel so lucky to have received so much attention and support from so many peers in such a short time; it feels like the whole world is helping me” (DB144).

### 5.3. Reference to the Other: A Buffer Space Beyond Real-World Dilemmas

Online expression became a key means for postgraduate students to explore ways to break through deadlocks in their supervisor–postgraduate relationships; digital communities thus became platforms for them to bear and transform their real-world challenges. Online narrative was not merely a simple emotional outlet but rather a core practical process through which postgraduates continuously interpreted, reflected on, and reconstructed the chaotic and disordered dilemmas of the supervisor–postgraduate relationship through interaction. Online narrative was not only the externalization and expression of emotions but also a meaning-making process in which postgraduate students actively sorted through their dilemmas, integrated their cognition, and sought understanding, helping them establish an internal order amidst disordered real-life experiences. Through peer interactions within discussion groups, postgraduates could receive emotional comfort, learn from others’ experiences, and have their questions answered. This created a psychological buffer against real-world supervisor–postgraduate conflicts, alleviated negative emotions, and enabled them to explore viable strategies for overcoming these challenges.

#### 5.3.1. Experience Sharing: “The Postgraduate Student Survival Guide”

The interpersonal experiences and relationship-adjustment strategies that postgraduate students accumulated through supervisory interactions represented highly valuable practical knowledge (Vähämäki et al., 2021). Online narrative provided postgraduates with a medium for textual expression, enabling them to transform scattered and fragmented individual challenges into coherent, shareable texts. Through peer dialogue, they interpreted, validated, and integrated these experiences, solidifying them and constructing meaning from the individual to the collective level, ultimately distilling them into collective knowledge that could be communicated, referenced, and passed down. Narrative sharing facilitated the dissemination of individual experiences within the community, continuously enriching the digital community’s practical knowledge base. Some members jokingly referred to this as the “Postgraduate Student Survival Guide.” One participant reflected: “Having this group makes me feel very grounded. Here I have not only received a lot of advice but also made many friends” (DB198). Other postgraduate students could learn coping strategies from narratives of similar experiences, thereby reducing the trial-and-error costs associated with managing supervisor–postgraduate relationships and improving both the efficiency of matching and the satisfaction with the relationship. For the narrators themselves, the process of organizing fragmented experiences into coherent narratives and expressing them publicly constituted a structured reflection on complex supervisor–postgraduate experiences and a process of self-meaning construction facilitated by online narrative. This narrative reflection helped individuals better understand the challenges others face in supervisory relationships and also fostered critical thinking, enabling them to objectively review their own experiences from the perspective of a “veteran.” One member observed: “Before getting to know everyone here, I always thought I was alone. I never imagined there were so many peers facing similar issues in their interactions with their supervisors” (DB212). This, in turn, extended to rational examination of deeper issues such as the training system and the rights and responsibilities of supervisors. Some participants moved beyond individual experiences to systemic concerns: “What saddens me most is that so many classmates are feeling helpless. This reflects the inadequacies of the current postgraduate training system and the lack of transparency in university management mechanisms” (DB051). “The current supervisor system grants supervisors too much power, which is very unfriendly to students” (DB077). At the same time, experience sharing through narrative conveyed postgraduates’ attitudes and strategies for actively addressing supervisor–postgraduate relationships. Through group interaction, a stable and positive shared understanding was formed, achieving collective meaning-making at the community level and fostering a supportive community atmosphere. Influenced by this atmosphere, postgraduates deeply entrenched in supervisor–postgraduate dilemmas became more willing to share their personal experiences, express genuine emotions, and proactively seek ways to improve problematic relationships, thereby creating space for self-regulation that facilitates the healthy development of these relationships. One postgraduate student commented in the “Advice from Experienced Members” section: “The original poster’s sharing was very helpful. I have decided to have a similar conversation with my supervisor” (DB130). In summary, positive community interactions centered on online narrative and experience sharing not only served as a vital channel for postgraduate students to process their emotions and resolve conflicts within the supervisory relationship but also provided deep-rooted support for the stable and healthy development of these relationships.

#### 5.3.2. Problem Solving: “Self-Helpers in Supervisor–Postgraduate Conflicts”

Online discussion forums provided postgraduate students with an immediate and extensive channel for seeking assistance. The flexible and convenient social nature of asynchronous forums could effectively increase the likelihood of interaction among students (Koskey & Benson, 2017). By engaging in peer communication through written expression, postgraduates could quickly access knowledge resources and practical experience within their field while also receiving academic and emotional support through mutual assistance. This, in turn, helped them develop a clearer and more rational understanding of their challenges and enhanced their level of cognitive engagement (Kipling et al., 2023). This information exchange mechanism, grounded in narrative practices, provides online forum participants with a space for free communication, fostering information exchange and peer interaction (Adraoui et al., 2017). Consequently, it alleviated the sense of isolation associated with supervisory challenges, significantly shortened the problem-solving cycle, and mitigated the long-term accumulation of negative emotions related to the supervisor–postgraduate relationship. Specifically, when postgraduates entered discussion groups under the influence of negative emotions stemming from an imbalanced supervisor–student relationship, they often expressed themselves through narratives that reflect genuine emotions such as anxiety, tension, and helplessness. This was not only a manifestation of “self-rescue” in the face of isolation and helplessness within real-life dilemmas but also reflected their inner desire to seek understanding, improve the supervisor–postgraduate relationship, and resolve practical difficulties through narrative-based help-seeking. In this emotional state, postgraduate students tended to be more cautious in recounting facts and expressing confusion, fearing that their views might be overinterpreted and lead to misunderstandings. As one poster noted: “I am not blaming my supervisor; I just want to find an appropriate way to solve the problem” (DB090). Through candid narrative, sharing their struggles, and seeking advice, postgraduates received substantial help from their peers’ empathy, communication, and encouragement. This sense of shared struggle was echoed in another comment: “Reading others’ stories helped me see that I was not alone in these struggles” (DB195). In these narrative interactions, where they were listened to, understood, and supported, they alleviated their psychological burden. Through ongoing interaction, they gradually developed a rational understanding of their predicament, reconstructed their personal sense of meaning, and strengthened their confidence to confront and resolve problems. The reply came back with renewed determination: “Everyone’s suggestions are so comprehensive; I will definitely do my best to try them out” (DB247). At the same time, this study found that postgraduates who successfully overcame their difficulties proactively shared their experiences, completing a narrative identity shift from “help-seeker” to “supporter.” The shift from recipient to giver was captured in one final post: “Anyone who needs help can send me a private message. Those who have been caught in the rain always want to hold an umbrella for others” (DB044). This mutual aid process, centered on online narratives, not only continuously enriched the community’s practical knowledge base but also fostered a warm and inclusive supportive atmosphere. It transformed individual insights into shared collective experiences, playing a significant role in resolving supervisory conflicts, repairing relationships, and fostering a positive ecosystem of interaction.

### 5.4. Invisible Intimacy: The Extension of Presence in Real-Time Interactions

Online interactions created an open, cross-temporal dialogue space for postgraduate students (Yoel et al., 2023), providing a safe environment for individuals facing supervisory dilemmas to express themselves and share their thoughts. Narrative interactions among peers broke the cycle of solitary emotional exhaustion, allowing postgraduates’ confusion and feelings to be seen and understood. Individuals’ originally fragmented, private, and difficult-to-articulate supervisory experiences coalesced into a group consensus through peers’ empathetic communication and interpretive integration, thereby generating meaning at the collective level. While individuals soothed their emotions and refined their self-awareness through acceptance and support, the group formed stable emotional bonds through the process of sharing experiences, bearing emotions together, and jointly interpreting them. The sense of presence and emotional intimacy fostered by online interaction effectively alleviated the loneliness and helplessness postgraduates feel when facing supervisory conflicts alone. The emotional comfort and practical support provided by group companionship also served as a vital foundation for postgraduates to resolve supervisory dilemmas and restore their inner sense of order.

#### 5.4.1. Community Interaction: “No One Is an Island”

Characterized by open expression and the display of individuality, social media enabled individuals with similar experiences and shared concerns to transcend geographical and social boundaries, spontaneously forming interactive communities focused on specific issues (Boyd & Ellison, 2007). When postgraduate students engaged in community interaction through online discussion groups, they were essentially transforming their real-life experiences and inner feelings within the supervisor–postgraduate relationship into a public narrative practice that could be shared, discussed, and responded to. This narrative practice also became a crucial process for individuals to construct their own sense of meaning. Every post, comment, and interaction extended individual experience into collective experience; every instance of understanding and comfort strengthened the emotional bonds among members. One member expressed this sense of belonging: “Here I have found a sense of security and belonging; venting with everyone makes me feel so much better” (DB068). Ongoing dialogue centered on supervisory issues could rapidly bring together group members in similar situations, allowing individuals who were once isolated and helpless to find peer recognition through interaction and to confirm the validity of their own feelings through emotional resonance. This led to the formation of an emotional community where members protect and comfort one another, thereby achieving an initial generation of group meaning: “The internet has connected us; even understanding, guidance, and encouragement that seem out of reach ensure we are not isolated islands” (DB045). Spontaneous online communities centered on the supervisor–student relationship featured clear focal points and strong group cohesion, authentically documenting the real-life circumstances, emotional states, and expectations of postgraduates within these interactions. Through long-term, open community interaction, members learned from each other’s experiences, shared strategies, and offered mutual encouragement, gradually constructing a group-specific cognitive framework, coping logic, and mutual aid paradigm. Individuals no longer bore academic confusion and interpersonal pressures alone; instead, they transformed personal dilemmas into collective public issues, broadening their understanding through exchange and finding solutions by drawing on others’ experiences. One poster remarked: “Before getting to know everyone here, I always thought I was alone; I never imagined there were so many peers facing similar issues in their interactions with their supervisors” (DB092). Another poster stated: “After joining the discussion group, I learned a lot about how to interact with my supervisor, things I did not know before joining” (DB027). Through ongoing narrative interactions, individual experiences were validated, supplemented, and elevated; fragmented emotional experiences gradually coalesced into shared values and collective resilience, achieving both the refinement of individual cognition and the construction of collective meaning at the community level.

#### 5.4.2. Relationship Expansion: “We Are All Part of the Mainland”

Bound by shared experiences, emotional resonance, and the exchange of insights, the online interactions of postgraduate students within discussion groups naturally evolved into stable and reliable social connections. Online social interaction broke free from the constraints of traditional interpersonal relationships, which were often defined by geographical proximity, academic background, or supervisor–postgraduate ties. Centered on the shared theme of the “supervisor–postgraduate relationship,” it reconnected individuals who were previously scattered and unfamiliar with one another, building a new social circle grounded in understanding, trust, and support. The trust was mutual, as one member observed: “Everyone here is sincere; what I say here won’t be misunderstood” (DB136). Here, interactions were not driven by utilitarian goals but were based on sincere expression and empathetic companionship (Trespalacıos & Urıbe-florez, 2020). Postgraduates were able to let down their guard and communicate openly, releasing emotions and sharing their struggles in a safe environment: “Having this group makes me feel very grounded; here I have not only received a lot of advice but also made many friends” (DB248). Within this highly inclusive community space, individuals could clearly feel accepted, affirmed, and supported, thereby gaining a strong sense of security and identity: “Here I have found a sense of security and belonging; venting with everyone makes me feel so much better” (DB079). Another participant stated: “The unconditional affirmation and support from my peers are enough to form a series of islands, supporting me after so long adrift” (DB026). These heartfelt positive experiences continuously strengthened the emotional bonds between individuals and the group, transforming fleeting online interactions into lasting emotional dependencies and fostering collective meaning-making. More importantly, the relationships derived from discussion groups were not confined to the virtual space but continued to expand outward as trust deepened and needs evolved. The move beyond the screen was also noted: “Later, a few of us who got along well created a smaller group and started having meals together in real life” (DB167). Beyond the original discussion groups, postgraduate students spontaneously formed smaller, closer-knit communities, extending the understanding, companionship, and support forged online into deep, daily interactions. This expansion of relationships, moving from the inside out, from superficial to deep, and from virtual to real, made the emotional support built on shared experiences more stable and enduring. This process not only enriched postgraduates’ social support networks but also enabled individuals to continually refine their perceptions and adjust their mental states through sustained companionship and interaction. Under the dual influence of individual sensemaking and collective value identification, it achieved a mutually supportive relationship between individual growth and group symbiosis.

## 6. Discussion

### 6.1. Situations of Uncertainty and Cognitive Gaps: Why Postgraduate Students Turn to Online Narratives

Drawing on the “situation-gap-use” framework of sensemaking theory, the challenges that postgraduate students encounter in real-world supervisor–postgraduate interactions constitute “situations of uncertainty” in the process of meaning construction. Research indicates that tensions in the mentor–student relationship often stem from a deep-seated asymmetry. When supervisors and postgraduates fail to communicate clearly about their respective role expectations, preferred modes of interaction, and standards for feedback, a systematic misalignment of expectations is likely to occur (Golde, 1998, 2000; Barnes & Austin, 2009). This misalignment, in turn, affects the quality of the relationship and student development. In fact, such mismatches are not merely isolated communication errors; they are rooted in the social structure and cultural organization of postgraduate education. Information scarcity, a lack of community, and the ineffective transmission of tacit knowledge collectively undermine students’ persistence and their sense of academic achievement (Lovitts, 2001). When mutual expectations remain persistently misaligned and effective communication and mediation mechanisms are absent, postgraduates develop a pronounced cognitive gap, which also extends to their emotional and behavioral states. They find it difficult to fully articulate their authentic experiences in formal settings and struggle to obtain sufficient understanding and support from their surroundings. Power imbalances make it hard for postgraduate students to voice their concerns proactively, while the absence of conflict resolution channels allows problems to accumulate without being addressed. It is precisely within this institutional silence that postgraduates’ genuine concerns have long been suppressed, leaving them with no outlet for expression and no one to listen. Online narratives have become a crucial space for postgraduates to turn to precisely because they offer a relatively safe outlet for these suppressed concerns. In anonymous or semi-anonymous online communities, postgraduates can detach themselves from real-world power dynamics and social constraints, presenting their predicaments and expectations in a more direct and authentic manner. In other words, online narratives not only expose the phenomenon of expectation mismatch but also reveal a deeper issue. When formal institutional arrangements fail to accommodate students’ genuine voices, students carve out their own channels of expression. Postgraduate students turn to digital communities precisely to bridge the cognitive gap, a gap that has been deepened by institutional silence, through peer-based experiential references and emotional resonance.

### 6.2. Integration of the Four Dimensions of Meaning: How Online Narratives Help Postgraduate Students Construct Meaning

The four themes, namely identity formation, emotional interaction, experience sharing, and social connection, correspond to four dimensions of meaning that postgraduate students acquire in the process of meaning construction: existential meaning, emotional meaning, functional meaning, and social meaning. These four dimensions are not isolated; rather, they form an integrated whole in which they mutually support and intertwine with one another, collectively constituting the meaning-construction system for postgraduates facing academic guidance dilemmas.

First, existential meaning helps postgraduate students answer the question “Who am I?” Through online narratives, postgraduates achieve self-anchoring amidst uncertainty. The construction of postgraduates’ academic identity is a challenging process of socialization (Gardner, 2008; Weidman et al., 2001). They must strike a balance between rigorous academic demands and their personal lives, yet often lack sufficient recognition and support in reality (Adarlo et al., 2025). In the face of this dilemma, online narratives provide a digital extension for postgraduate students’ identity construction. When support from real-world supervisors is insufficient, peers’ online narratives serve as identity references, enabling postgraduates to affirm their position within the academic field through continuous writing and peer interaction.

Second, emotional meaning concerns the placement of individual emotions and empathic identification, responding to the inner need to feel “understood.” In digital communities, individuals’ repressed emotions find resonance and response at the group level, transforming isolated emotional experiences into collectively shared resources of meaning. Research indicates that postgraduate students’ mental health issues are closely linked to the quality of their supervisory relationships, and the lack of emotional support is a significant source of psychological stress for them (Levecque et al., 2017; Ogilvie, 2020). With their anonymity and low-threshold social interaction, online communities provide a safe space for emotional expression, helping postgraduates bridge the emotional-cognitive gap.

Third, functional meaning focuses on coping with real-world challenges and acquiring practical skills, addressing the practical confusion of “What should I do?” By sharing experiences and seeking guidance, postgraduates transform personal confusions into public texts for discussion. Through peer dialogue and validation of experiences, these texts crystallize into a shared knowledge base within the community. Faced with challenges in the supervisory relationship, postgraduate students often feel “unsure of what to do” (Phillips & Johnson, 2022; Y. Wu et al., 2025). The peer experience-sharing provided by digital communities constitutes a “horizontal learning” model (Boud & Lee, 2005; Wilson et al., 2023), which compensates for the shortcomings of in-person guidance and enriches the pathways of postgraduates’ academic socialization.

Finally, social meaning is grounded in group belonging and relational connections, addressing the value-based inquiry of “With whom do I walk?” The online interpersonal relationships formed in discussion groups gradually extend into real-world settings, thereby creating a stable, long-lasting peer support system. Extensive research indicates that postgraduates’ sense of belonging is closely linked to their academic persistence and mental health. When postgraduate students perceive a strong sense of community belonging, they not only exhibit lower levels of anxiety and depression but also demonstrate greater willingness and motivation to persist in their studies (Farra et al., 2025; Pyhältö et al., 2009). Conversely, a lack of belonging increases psychological distress and the risk of withdrawal among postgraduates (Tommasi et al., 2022). When the physical educational environment lacks systematic and effective in-person support communities, the social connections provided by digital communities break through the constraints of traditional interpersonal interactions, offering postgraduates a sustainable sense of belonging and a support system.

### 6.3. Institutional Compensation: Online Narratives as an Alternative Support System

By integrating these four dimensions of meaning, a core proposition can be distilled: online narratives serve as a “compensatory system” for postgraduate students in the absence of institutional support. From an institutional perspective, the current coordination mechanisms for supervisor–postgraduate relationships in universities remain underdeveloped and lack systematic institutional support. Driven by university performance accountability systems, these relationships operate within a dual principal-agent model. Coordination between supervisors and postgraduates largely relies on individual-level negotiation. However, constrained by power imbalances and institutional rigidity, such negotiations often fail to effectively resolve conflicts and may even exacerbate the deterioration of the relationship (J. Zhang, 2024). From the perspective of interactive processes, the inherent power asymmetry and institutional constraints within the supervisory relationship, coupled with divergent expectations and poor communication between supervisors and students, further intensify the risk of relationship deterioration (J. Han & Jin, 2025). According to organizational support theory, perceived organizational support influences employees’ commitment to and behavior within the organization (Eisenberger et al., 1986). When individuals perceive insufficient organizational support, they turn to other social relationships to seek alternative sources of support (Rhoades & Eisenberger, 2002). Digital communities can thus be viewed as alternative social support systems proactively constructed by postgraduate students in contexts where institutional support is lacking. This finding reveals a profound paradox in the development of supervisor–postgraduate relationships in the digital age. Online narratives serve as a “self-rescue” channel for postgraduates in the absence of institutional support, while simultaneously exposing systemic shortcomings in offline educational management. The proliferation of online narratives should not be simplistically viewed as “internet dependency” among postgraduate students; rather, it must be understood as a compensatory response to institutional gaps. When postgraduates cannot obtain identity validation, emotional resonance, experiential references, or social connections in the real world, they construct an alternative support system within virtual spaces. This system provides them with vital psychological support and experiential resources. However, overreliance on online narratives may also lead postgraduates to abandon effective communication in the real world, miss opportunities to repair relationships, and create a tension between “substitute support” and “real-world communication.” How to strike a balance between these two remains a pressing practical issue that must be addressed in the development of supervisory relationships in the digital age.

### 6.4. Research Implications

In terms of theoretical contributions, this study further reveals the intrinsic logic of online narratives as a practice of meaning construction. While traditional research on meaning construction has largely focused on individual information behaviors and cognitive processes, this study extends the scope to include group emotional interactions and collective meaning generation. It finds that meaning-making in online communities exhibits a cyclical “individual–group–individual” pattern. Individual narratives trigger group resonance; group wisdom feeds back into individual cognition; and individuals anchor and reconstruct their identities through continuous interaction. Building on this, the study further distills four dimensions of meaning-making through online narratives, namely existential meaning, emotional meaning, functional meaning, and social meaning. This provides an integrated analytical framework for understanding the mechanisms of meaning construction among individuals facing dilemmas in the digital age. Furthermore, the study reveals the underlying logic of online narratives as “institutional compensation,” expanding the research perspective on the supervisor–student relationship from a “teacher–student binary interaction” to a three-dimensional framework of “institution, individual, and technology.” While these theoretical contributions are derived from a Chinese case, they offer transferable analytical tools that may inform international scholarship on postgraduate student well-being, digital peer support, and the evolving dynamics of supervisory relationships in the digital age.

The problems reflected in online narratives, such as expectation misalignment, insufficient emotional support, and power asymmetry, are widely documented across different national contexts (Moshtari & Schleper, 2025; Sidhu et al., 2014; Thomas et al., 2024). However, the practical implications below are specifically tailored to the Chinese higher education landscape, given its distinctive institutional features, such as the shimen (mentor–apprentice) tradition (L. Liu & Dong, 2025; Z. Liu et al., 2026), the prevalent project-based utilitarian academic culture, and the underdeveloped on-campus grievance and mediation mechanisms (H. Ma et al., 2023). These are, therefore, offered as context-sensitive strategies for Chinese universities. Nevertheless, the theoretical mechanisms they draw upon may offer valuable reference for other national contexts grappling with similar challenges in postgraduate supervision. From a practical standpoint, the supervisor–postgraduate relationship in the digital age exhibits distinct dual characteristics. Online narratives serve as a vital channel for postgraduates to address real-world challenges and compensate for insufficient formal support, while also vividly reflecting the structural shortcomings in offline supervisory governance and educational management within universities. Based on the aforementioned theoretical deductions and empirical observations, this study offers the following practical implications.

First, in-depth research into postgraduate student online communities is needed to identify latent issues that are difficult to detect through offline management alone. Universities can establish specialized research institutions or project teams to identify blind spots and challenges in offline educational management that are revealed through online narratives, such as communication barriers between supervisors and students and emotional detachment. Mismatched expectations resulting from poor communication are the primary cause of conflicts in the supervisor–student relationship (Inter-University Doctoral Administrative Committee [IDAC], 2022). Accurately identifying these latent issues can provide a practical basis for formulating scientifically sound and effective intervention strategies.

Second, digital media literacy should be incorporated into training systems covering both administrators and faculty supervisors. Many supervisors view students’ online complaints as emotional venting or personal grievances, leading them to respond with criticism or defensiveness. Training should help supervisors understand that online narratives represent students’ “self-rescue” behavior in the absence of institutional support, serving as a vital means of emotional release and meaning-making. This understanding should guide supervisors to respond to students’ difficulties with a more open and empathetic attitude.

Third, offline coordination mechanisms for supervisory relationships should be established to effectively bridge online and offline interactions. Universities must recognize the value of online narratives as a window into student concerns and simultaneously establish offline coordination mechanisms. Through activities such as workshops, seminars, and training in social and emotional skills, universities should provide postgraduate students with safe and open platforms for communication. Research indicates that academic institutions generally fail to provide supervisors with sufficient formal channels to reflect on their supervisory practices (Hammond et al., 2010). Given this deficiency, universities should provide supervisors with ongoing training and professional development opportunities (Buirski, 2022; Haley et al., 2025) to help them enhance their supervisory skills and better resolve conflicts and challenges in the supervisory relationship.

Ultimately, institution-specific support networks should be developed to provide postgraduate students with independent and impartial channels for seeking assistance. A single supervisor is unlikely to meet students’ diverse needs; postgraduates require support through collaborative supervision by multiple supervisors (Skarakis-Doyle & McIntyre, 2008). Universities can establish interdisciplinary supervisory support networks, creating platforms such as “interdisciplinary supervisor pools” and “peer support groups.” In reality, such independent and neutral channels for assistance are generally lacking, which is precisely why postgraduates turn to online communities to seek emotional comfort and support.

### 6.5. Limitations

While the findings offer meaningful insights, several limitations warrant consideration. First, the empirical data were drawn exclusively from a single Douban group, which limits the generalizability of the findings to other online communities or platforms. Second, as with any netnographic study based on publicly available online data, the identity of the participants cannot be verified with certainty. While the linguistic context, discussion topics, and group description strongly suggest that the majority of contributors are postgraduate students enrolled in Chinese universities, Douban does not require users to disclose their institutional affiliation or nationality. Future research could complement online textual analysis with offline interviews to triangulate the findings. Third, the findings are situated within the specific cultural and institutional context of Chinese postgraduate education. The narrative patterns, emotional dynamics, and meaning-making processes identified here may not be directly transferable to other national or cultural contexts. Cross-national comparative research is needed to examine the applicability of the four-dimensional meaning-making framework beyond China. Despite these limitations, the study provides a rich, contextually grounded account of how postgraduate students use online narratives to make sense of their supervisory experiences, offering both theoretical and practical insights for understanding supervisor–postgraduate relationships in the digital age.

## 7. Conclusions

As a complex and multidimensional system of social relationships, the supervisor–postgraduate relationship is interwoven with multiple influencing factors, including power structures, emotional bonds, and institutional constraints. This study focuses on the Douban group “Has My Supervisor Cared for Me Today?” and employs online ethnography and thematic analysis to systematically investigate the thematic dimensions of meaning in postgraduates’ online narratives about the supervisory relationship.

The findings reveal that postgraduate students have developed a diverse and clearly stratified system of narrative expressions regarding the supervisor–postgraduate relationship within this anonymous digital community. These narratives primarily crystallize into four core dimensions: identity formation, emotional interaction, experience sharing, and social connection. Online narratives provide postgraduate students with an opportunity to reflect on their supervisory experiences and examine their personal growth, enabling them to continuously construct and refine their academic identities through the organization of experiences and group reference. The relaxed and anonymous atmosphere of online expression also allows postgraduates to release emotional pressures that are difficult to articulate in real-life contexts, gaining emotional connections and spiritual solace through peer empathy and understanding. At the same time, individual and scattered experiences of supervisor–postgraduate interactions gradually crystallize, through narrative sharing, into peer-based practical knowledge that can be referenced and emulated, forming a collective knowledge paradigm with transmissible value. Furthermore, sustained interaction within the community breaks down the boundaries imposed by academic affiliations, supervisor–postgraduate ties, and geographical locations, establishing spiritual connections and interpersonal networks that transcend time and space. This resonance within the virtual community gradually extends to real-life relationship building, fostering a robust peer support community. For postgraduate students, online narrative sharing and community interaction have become crucial processes for bridging cognitive gaps and generating diverse forms of meaning. Existential, emotional, functional, and social meanings do not progress in a linear fashion; rather, they are intertwined and mutually constitutive. Together, they follow a cyclical logic of individual narrative, group resonance, and individual reconstruction, helping postgraduate students achieve identity anchoring, emotional healing, experience acquisition, and a renewed sense of belonging through continuous interaction. Furthermore, the rise of online narrative is, in essence, a compensatory self-help channel formed by postgraduate students in response to insufficient institutional support. It not only provides a private space for individuals to resolve conflicts with their supervisors and seek emotional support but also vividly exposes the structural shortcomings in universities’ offline supervisory governance, emotional care, and conflict resolution mechanisms.

Conclusively, in the digital age, the supervisor–postgraduate relationship has transcended the scope of traditional offline binary interactions. Online narratives have become a crucial window for gaining insights into postgraduate students’ authentic experiences with their supervisors and for identifying latent conflicts within these relationships. This study has clarified the core thematic dimensions of meaning in online narratives about the supervisor–postgraduate relationship, thereby enriching the digital perspective in research on these relationships. It also provides theoretical insights and practical guidance for universities to optimize their supervisory and educational management systems and to establish an integrated support service framework that combines online and offline efforts.

## Figures and Tables

**Table 1:** Coding table of meaning in online narratives of supervisor–postgraduate relationships.

Theme	Sub-Theme Coding	Open Coding	Reference Points
**Identity** **Formation**	Identity Construction	Self-positioning	58
		Role Interaction Experience	64
	Self-Reflection	Growth Review During Enrollment	128
		Academic Record Reflection	88
**Emotional Interaction**	Emotional Expression	Positive Emotion Sharing	149
		Negative Emotion Release	127
	Emotional Resonance	Presentation of Emotional Experiences	114
		Evoking Emotional Responses	166
**Experience Sharing**	Experience Sharing	Exchange of Similar Experiences	115
		Learning Practical Strategies	107
	Problem-Solving	Immediate Problem Identification	101
		Collective Wisdom Provision	123
**Social Connection**	Community Interaction	Cognitive Integration into the Community	65
		Building Support Networks	73
	Relationship Expansion	Sustaining Online Relationships	65
		Extending Subgroup Relationships	42

## Data Availability

The data that support the findings of this study are available from the corresponding author upon reasonable request.
